# Redefining intestinal immunity with single-cell transcriptomics

**DOI:** 10.1038/s41385-021-00470-y

**Published:** 2021-11-30

**Authors:** Kylie Renee James, Rasa Elmentaite, Sarah Amalia Teichmann, Georgina Louise Hold

**Affiliations:** 1 Garvan-Weizmann Centre for Cellular Genomics, Garvan Institute of Medical Research, Sydney, NSW 2010 Australia; 2 School of Medical Sciences, University of New South Wales, Sydney, NSW 2006 Australia; 3 Wellcome Sanger Institute, Wellcome Genome Campus, Hinxton, CB10 1SA UK; 4 Theory of Condensed Matter Group, Cavendish Laboratory/Department of Physics, University of Cambridge, Cambridge, NSW CB3 0HE UK; 5 University of New South Wales Microbiome Research Centre, Sydney, NSW 2217 Australia

## Abstract

The intestinal immune system represents the largest collection of immune cells in the body and is continually exposed to antigens from food and the microbiota. Here we discuss the contribution of single-cell transcriptomics in shaping our understanding of this complex system. We consider the impact on resolving early intestine development, engagement with the neighbouring microbiota, diversity of intestinal immune cells, compartmentalisation within the intestines and interactions with non-immune cells. Finally, we offer a perspective on open questions about gut immunity that evolving single-cell technologies are well placed to address.

## Introduction

The intestinal tract contains a plethora of immune cells that are essential for normal physiology and defending the body against potential pathogens, but may also contribute to disease when their responses are exacerbated. Since the recognition of a localised intestinal immune system in 1919^1^, evolving technologies and experimental systems have helped refine our understanding of this complex cellular network.

The invention of single-cell RNA sequencing (scRNAseq) in 2009^2^ has revolutionized the field of immunology, revealing an unappreciated complexity of immune cell subsets, identifying new cell types and states, redefining cellular ontogeny and enabling inference of cell fate trajectories and function^3,4^. ScRNAseq is able to piece together existing knowledge of cell markers, ontology and interactions into an integrative picture of the building blocks of human tissues. Applied to human mucosal immunity, scRNAseq is particularly powerful as it allows for systematic analysis of cells within these complex and highly-immunologically active tissues, thereby making the most of small and often difficult to obtain clinical samples. Although transcriptional expression is not a perfect readout of protein expression^5^, scRNAseq allows for the hypothesis-generating phase of research to begin with and be guided by tissue-specific clues. Targeted experiments in model systems can then be used to support findings and test biological mechanisms. In this way and spurred on by the conception of the Human Cell Atlas (HCA) initiative in 2016, scRNAseq has been applied with great effect to several human barrier tissues including skin^6^, reproductive organs^7,8^ and mouth^9^, and recently in the context of SARS-CoV-2 infection^10–14^.

In this review, we focus on the immune system of the intestinal tract and specifically discuss how single-cell transcriptomics has advanced knowledge in this field. We provide an introduction to scRNAseq methods and analysis tools with particular use in this area and highlight studies that have shed light on the origins of intestinal immunity, cell diversity and plasticity, interactions with non-immune cells and compartmentalisation within the tissue architecture.

## ScRNAseq approaches to studying intestinal immunity

The scRNAseq field is rapidly evolving, with the number of cells captured per experiment now in the millions. Approaches to single-cell profiling intestinal tissues vary between studies and depend on tissue availability and biological questions being asked. Current studies on intestinal immunity compare cells of healthy or IBD patients^15–18^, focus on regional differences^19–21^ or investigate intestinal development^22–24^ applying either in-depth or high-throughput methods, and increasingly combining other technologies such as V(D)J sequencing and spatial transcriptomics to better understand cell heterogeneity, lineage relationships and spatial locations in tissue^20,21,24^. Below we outline the current and emerging technologies and analysis tools for studying intestinal immunity.

### ScRNAseq platforms

**Fig. 1:**
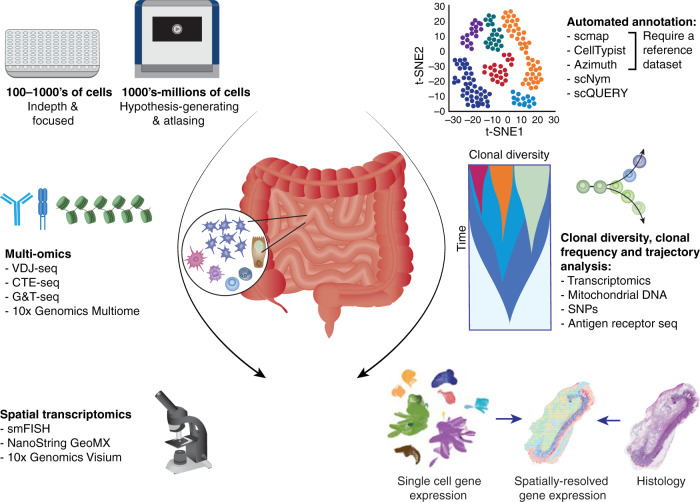
ScRNAseq approaches and analysis tools for studying intestinal immunity. Left: ScRNAseq, multi-omics and spatial platforms; Right: mirrored analysis tools for analysing resulting data. sm-FISH: single molecule fluorescence in situ hybridization; SNPs: single nucleotide polymorphisms.

There is a range of scRNAseq methods available with different benefits for studying mucosal immunology^25–28^. Platforms relying on the isolation of dissociated cells by fluorescence-activated cell sorting (FACS) or microfluidic devices include STRT-seq^29^, CEL2-seq^30^, MARS-seq^31^ and SMART-seq2-3^32–34^. Use of FACS provides auxiliary information of proteins targeted by a select panel of fluorophore-tagged antibodies and can help unite transcriptional profiles to traditional cell type identities. These methods are lower in throughput due to limited capture sites, ranging from 100–1000’s of cells per experiment (Fig. 1). A benefit of SMART-seq methods in particular is that they provide sequencing of full-length transcripts such that highly variable transcripts including B and T cell receptors (BCR and TCR respectively) are automatically included, and they generally have greater coverage of the transcriptome compared to high-throughput approaches detailed below. Together, these approaches are especially useful for in-depth and targeted analysis of immune cell types.

High-throughput approaches rely on capture through microfluidic devices of single cells in water droplets in an oil phase (10x Genomics Chromium Gene Expression, Drop-Seq^35^ and inDrops^36^) or in microwells (Seq-Well S^3^ ^37^ and STRT-seq-2i^38^). These methods tag either the 3′ or 5′ end of mRNA, incorporating a unique molecular identifier and applying a cell-specific barcode early after cell capture. Total mRNA can then be pooled for downstream library preparation allowing for processing of 1000’s-millions of cells per experiment (Fig. 1). A major drawback of tagging either end of the mRNA is that highly variable transcripts such as splice variants and antigen receptors are not reliably captured. However, targeted amplification of TCRs and BCRs can be included as an additional step for the 10x Genomics Chromium 5′ platform. The high-throughput, less targeted nature of these methods makes them ideal for tissue atlasing or hypothesis-generating experiments.

Recent advances in the realm of multi-omics technology, in which multiple cell features are simultaneously measured, are building on scRNAseq methods to also shed light on diversity in cell genotypes, transcriptional regulation and protein expression. These approaches include genotyping plus transcriptomics available as G&T-seq^39^, chromatin-accessibility with transcriptomics available via the 10x Genomics Multiome ATAC + Gene Expression platform and targeted protein quantification plus transcriptomics available as CITE-seq^40^ or REAP-seq^41^. Spatial transcriptomics is another rapidly evolving area and has already provided spatial context for cell identities or cell-cell interactions identified from scRNAseq studies of the gut mucosa^24,42^. Available platforms include 10x Genomics Visium and nanoString GeoMx Digital Spatial Profiler, with the former currently offering whole transcriptome capture of zones covering in the order of 10 cells, and the second providing simultaneous fluorescent imaging at single cell resolution and whole transcriptome profiling from tissue regions of interest.

### Analysis of scRNAseq data

Pre-processing and analysis of scRNAseq data from low- and high-throughput platforms follows the same general workflow and is detailed in a number of review articles and online tutorials^43,44^. In short, raw sequencing data undergoes read quality control, assignment to cellular barcode, mapping to a reference genome and read quantification to obtain a cell by gene matrix, and can be done in pipelines such as Cell Ranger^45^, indrops^46^, SEQC^47^, or zUMIs^48^. The data can then be handled with well documented computational packages as part of Seurat^49^, Scanpy^50^ and OSCA^51^ for quality control to remove empty droplets/poor quality cells, normalisation of the data and dimensionality reduction in preparation for visualisation. Downstream analysis of scRNAseq data typically involves cell clustering and cell type/state annotation, trajectory analysis^52^ and ligand-receptor expression analysis^53^. Manual cell type annotation of clustered scRNAseq data is an iterative and laborious process. The most recent advances in the scRNAseq analysis include the development of automated tools for this step in the analysis pipeline. Amongst these methods, recently reviewed by ref. ^54^, are correlation based methods that require a reference dataset (e.g., scmap, CellTypist^55^ and Azimuth^49^) and neural network based algorithms without a prior reference (e.g., scNym^56^ and scQUERY^57^) (Fig. 1). Reference-based methods are gaining popularity, but their output relies greatly on the relevance and quality of the cell type reference. For example, CellTypist^55^ provides a collection of comprehensive and carefully curated immune cell profiles from multiple organs suited for the annotation of human tissue immune cells. Trajectory analysis for tracing cell type development pathways or fate decisions is commonly carried out with packages including Monocle^58^, Wanderlust^59^ and Slingshot^60^. Each of these methods order individual cells based on gene expression along a pseudotime trajectory describing a transient continuous biological process. Alternative or complementary approaches to lineage tracing implement paired antigen receptor sequences, single nucleotide polymorphisms^61^ or variance in mitochondrial DNA^62^ as natural barcodes to track differentiation or migration of clonally related cells. RNA velocity analyses such as scVelo^63^ leverage splice variant information held within scRNAseq data to also map cellular response and developmental kinetics. Finally, recent tools to map cell signatures determined from scRNAseq data into spatially resolved transcriptomics data include cell2location (preprint available^42^) and within the Seurat framework.

## Development of intestinal immunity

### Formation of gut-associated lymphoid structures

Intestinal immunity is established during development in utero. Prior to the single-cell genomics era, survey of immune populations during human development has been challenging, owing to tissue access, and there has been limited knowledge about the populations and markers expressed at this stage. Single-cell transcriptomics have been instrumental in understanding the diversity of immune and non-immune populations in these precious human intestinal developmental tissues^22–24,64^.

**Fig. 2:**
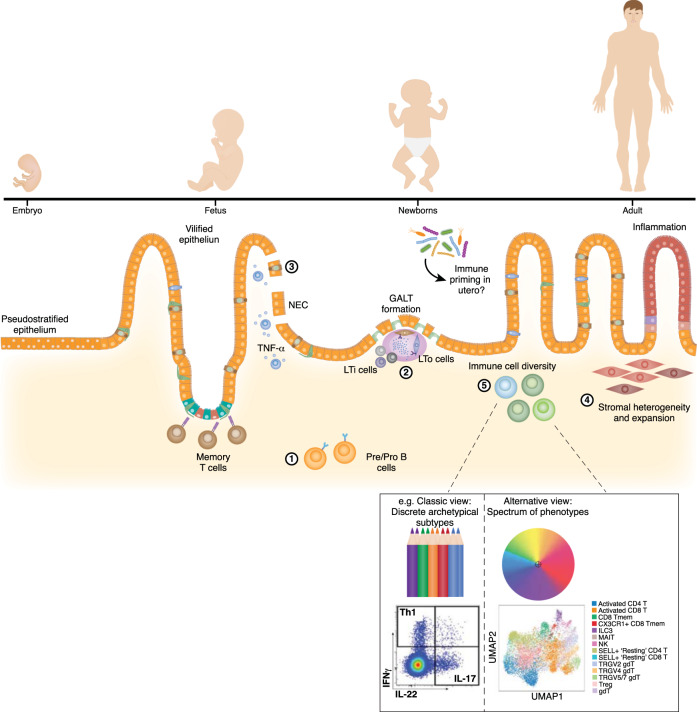
ScRNAseq advances in understanding mucosal immunity throughout life and during disease. (1) Building from observations of lymphocyte maturation in the prenatal intestines, single-cell studies have promoted the concept that T and B cell activation up to second-trimester development is in support of intestinal development rather than in response to microbial seeding^21,74–76^. (2) Key players in gut-associated lymphoid tissue (GALT) formation previously identified in developing mouse gut have been identified in scRNAseq studies of human fetal gut, with additional cell heterogeneity identified^21,24^. (3) TNF-α-producing CD4+ T cells resolved at single-cell level are enriched in the preterm intestines, likely contributing to epithelial damage observed in necrotising enterocolitis (NEC)^77^. (4) Intestinal stromal heterogeneity has been determined, and expanded subtypes in inflammatory bowel disease linked with pathology^16–18^. (5) Inlaid box depicts the classical idea of discrete immune cell subsets versus a spectrum of phenotypes determined through scRNAseq analyses^88^. LTi lymphoid tissue inducer, LTo lymphoid tissue organiser.

The intestinal immune system is supported and regulated by gut-associated lymphoid tissues (GALTs), including mesenteric lymph nodes, Peyer’s Patches (PPs) and cryptopatches. Despite obvious differences in gut lymphoid tissue size and location between species^65^, our understanding of the development of these structures has previously relied on experiments in animals^66^. In mouse studies, early GALT formation has been described to involve interactions between mesenchymal lymphoid tissue organising (mLTo), endothelial LTo (eLTo) and lymphoid tissue inducer (LTi; related to innate lymphoid cells (ILCs)) cells^66^. Interactions between these cell types are critical for recruiting and retaining immune cells at the sites of developing lymphoid structures. Through scRNAseq of human fetal gut samples, central players in secondary lymphoid organ formation have been resolved in humans, and their communication programs in initiating PP formation are defined from as early as 12 weeks post-conception (Fig. 2)^21,24^. In addition, multiple subsets with LTi characteristics have been identified, proposing differences between human and mouse development^21^. Importantly, by comparing single-cell transcriptional profiles, equivalent stromal populations are predicted to be involved in the formation of ectopic lymphoid structures during inflammatory bowel disease (IBD), suggesting reactivation of developmental programs to support intestinal inflammation^21,24^. Fawkner-Corbett et al. described a population of mLTo-like stromal cells (i.e., with *CCL19, CCL21* and *CXCL13* expression) with similarities to a subtype of stromal cells expanded in ulcerative colitis (UC)^17,24^. Taking advantage of recent spatial transcriptomics technology, the authors showed localisation of these cells and confirmed the likelihood of relevant cell-cell interactions in lymphoid follicle formation in situ^24^. This shows that the formation of secondary organs is not restricted to development and is required for proper maturation and response of the immune system.

### First encounters with the microbiota

We live in an era where the relationship between our immune system and microbes has never received such unprecedented attention. Characterising human-associated microbiotas and their role in health and disease has become the holy grail of current medicine. It is well-established that the human-associated microbiota contains a wide and complex community of microorganisms that is unique to individuals and constantly evolves in response to its environment^67,68^. Microbial dysbiosis is well recognised in diseases such as IBD, colorectal cancer, metabolic disorders and in conditions including pregnancy although mechanistic insight into the host:microbial relationship remains in its infancy^69^. Whether the interaction is between the host and its resident microbiota or a direct response to a specific infectious entity, microbes communicate with the host through attachment to mucosal surfaces, binding specifically to host receptors, production of metabolites such as short chain fatty acids and bile acids or adapting their growth and metabolism based on changes we make to their local environment. In parallel host immune responses attempt to continuously decipher between microbial friend or foe.

The question of when host–microbe interactions become established has become a topic of intense investigation, with the presence of microbiota during in utero development still highly debated. A recent study of the meconium microbiota in human neonates (at term) before birth, controlling for process/delivery mode-induced contamination indicated that microbial colonization most likely occurs either during birth via maternal seeding or post-birth via environmental seeding^70^. Conversely, microscopic images of bacterial-like structures with mucin threads within the gut lumen during the second gestational trimester provide compelling evidence for in utero seeding as well as aligning with other studies that detail in utero antigenic priming of the fetal immune system^71^. However, these studies are caveated with the potential of contamination with environmental sources of microbes (reviewed in ref. ^72^) making their physiological relevance questionable.

Nevertheless, priming of the immune system and unexpected activation of immune cells have been suggested to be linked to the early microbial colonisation in fetal organs, especially the gut^73^. In particular, multiple scRNAseq studies have shown that memory CD4+ and CD8+ T cells are present and clonally expanded in the intestines in the first and second trimester of development (Fig. 2)^74–76^. However, in a scRNA-seq study by Schreurs et al. fetal intestinal CD4+ T cells had a distinct gene expression profile from those in the post-natal intestine, and were characterized by high expression of genes regulating cell cycle, WNT signalling, and tissue development^77^. This supports the role of CD4+ T cells in fetal intestines promoting tissue development. A study using cytometry by time of flight (CyTOF) in combination with BCR sequencing showed that B cells are immature during second-trimester human development compared to those found in infants^75^. Our scRNAseq of human fetal intestines up to 17 weeks post conception also showed no evidence of B cell clonal expansion, class-switching or germinal centre formation^21^. Prenatal B cells may similarly be involved in development of lymphoid structures and have no need for class switching, while postnatal B cells undergo these events due to the presence of microbiome^75^. Through more precise analysis of cell phenotypes, these single cell studies promote the emerging concept that immune cell activation at least until second-trimester development is a product of their support of a highly controlled process of tissue generation rather than due to microbial seeding. Whether this is also the case in the third trimester of human development remains to be determined.

### Necrotising enterocolitis

It has been argued that the epithelial barrier in preterm infants is immature; unable to sustain the ensuing microbial colonisation due to epithelial leakiness. Necrotising enterocolitis (NEC) is a devastating intestinal disease that occurs primarily in premature infants, resulting in impairment of the epithelial barrier and in extreme cases causing intestinal perforation and tissue necrosis^78^. Studies have consistently highlighted differences in bacterial gut communities associated with NEC that result in an imbalance between pro- and anti-inflammatory gut immune mediators^79,80^. Work by Cho et al. used mouse models to highlight an imbalance within the adaptive immune system in the NEC intestinal environment typified by type 3/T helper (Th)17 polarization, with reduced Th1, Th2, and Treg responses^81^. These findings were further supported by scRNAseq studies showing preferential presence of TNF-α-producing CD4+ T cells in early intestinal development and an enrichment for these cells in the intestines of preterm infants with NEC (Fig. 2)^77^. The TNF-α overloaded microenvironment likely contributes to NEC-associated epithelial damage^77^. In addition to the effects of IL-10 in promoting self-renewal of stem cells, the potential of T cell cytokines IFNy, IL-17A and IL-13 in promoting differentiation of epithelial cells towards mature cell types has been shown in adult mice^82^. The capacity of T cells to inform epithelial cell differentiation and maturation provides the opportunity to harness this interaction in clinical practice for treatment of NEC and other gut disorders.

Bacterial dysbalance in the premature intestines is considered one of the key factors contributing to NEC. No single microbe has been identified as the mea culpa for NEC although increased abundance of Proteobacteria are frequently reported in NEC infants^83,84^. A recent study analysed microbial features predictive of NEC and identified Enterobacteriaceae overgrowth; including specifically Klebsiella—known to possess secondary metabolite gene clusters related to quorum sensing and bacteriocin production to be replicating more rapidly in the days prior to NEC diagnosis^85^. The transcriptional and proportional cell changes are likely reflected in these preterm infants and future single-cell studies will be instrumental in defining these changes.

## Heterogeneity and plasticity of intestinal immune cells

The immune system must exhibit diversity and plasticity to respond to the countless challenges incurred throughout life. The conventional approach to studying diversity in immune cells has been top down—focussing on a cell type and iteratively subdividing it into more distinct subsets based on marker gene expression. This approach has been essential in understanding the intestinal immune system, but relies on pre-selection of markers and is limited in resolving heterogeneity within distinct cell groups. A strength of scRNAseq is its ability to explore heterogeneity from the bottom up—dividing cells into distinct groups and then defining molecular profiles that best describe each population^4^. In this way scRNAseq has refined classical immune cell type labels, defined new populations and predicted the role of cell types and states in the intestinal immune system of both mice^86^ and humans^16,87–89^.

ILCs are innate immune cells that defend against both intra- and extracellular infections and are particularly abundant in mucosal tissues^90^. While they do not possess a functional TCR, they draw parallels in function and subtype classification with Th cells. ILCs are typically divided into 5 types- ILC1, ILC2, ILC3, natural killer and LTi cells^91^. A study by Muzzurana et al. compared sorted CD127+ ILCs from human blood, lung, colon and a past tonsil dataset^92^ using the Smartseq2 platform^87^. Adopting a bottom-up approach to classifying ILC subtypes, they performed unbiased clustering followed by differential expression analysis and correlation analysis on the pooled data. ILCs subdivided into 20 subsets, clustering largely by tissue origin and FACS phenotype. Highest ILC3-associated gene expression was detected in the colon as expected^90^, but also the highest degree of diversity covering a spectrum of signatures ranging from migratory (expressing *SELL*, *S1PR1*, *ITGAX* and *GPR183*) to activation and tissue residence (expression of *IL22*, *NCR2*, *GRM7* and *LTA4H*)^87^. ILC heterogeneity has similarly been shown with scRNAseq of mouse intestines^86^. To show the influence of the neighbouring microbiota on ILC signatures, the authors of this study treated mice with antibiotics prior to scRNAseq analysis. In antibody-treated mice, profiles of ILC1 and ILC2 more closely resembled ILC3 cells (with increased *Atf5*, *Cxcl9* and *Gpx1*) compared to mice with an intact microbiota^86^. This points towards ILC3 representing the “default” phenotype with environmental factors driving diversification.

Amongst the most diverse immune cells are CD4+ T cells, which have classically been partitioned into discrete subsets according to their expression of key transcription factors and cytokines (e.g., Th1 and Th2 cells expressing IFNy/TBET and IL4/IL5/IL13/GATA3, respectively). However, plasticity or merging between these subsets has been a frequent observation in mice and humans^93^. A scRNAseq study by Kiner et al. observed extensive heterogeneity and blended signatures of colonic T cells in specific pathogen-free mice^88^. In an attempt to drive Th differentiation they infected mice with *Citrobacter rodentium*, an inducer of Th17 cells (determined by IL-17 expression), *Heligmosomoides polygyrus* and *Nippostrongylus brasiliensis*, both inducers of Th2 (IL-5 and IL-13) responses or *Salmonella enterica*, a bacterial infection inducing Th1 (IFNγ) responses. While FACS of T cells from infected mice confirmed the expected skewing of Th differentiation, scRNAseq analysis and unbiased clustering separated cells by infection system rather than characteristic Th genes. Expression of canonical Th cytokines dominated opposing sides of the same clusters in their data, arguing against discrete subsets and in favour of a polarised continuum of Th phenotypes driven by the infection setting^88^ (Fig. 2, inlay). Other scRNAseq studies of acute immune responses in mice have reported skewed Th signatures including Th1 and Tfh in peripheral blood of mice infected with *Plasmodium*^94^, Th2 in lungs of mice exposed to dust mites^95^ and Th2 in spleen and lymph nodes of mice infected with *Nippostrongylus brasiliensis*^96^, but have also showed heterogeneity and blending of canonical marker genes between clusters. Spectrums of Th phenotypes have also been resolved at single-cell level within the human breast cancer tumour microenvironment^47^, asthmatic lung^97^ and in blood of SARS-CoV-2 infected individuals^98^.

ScRNAseq studies of human intestinal disease have similarly added to our extensive understanding of the diversity in T cell phenotypes and highlighted specific enriched populations of likely significance to pathology. One such study observed a Th17-like population of *CD4*+ *CD8*+ cells expanded in UC^16^. Given the known association between Th17 cells and IBD^99^, the authors hypothesised the role of the Th17-like cells in driving inflammation, although this remains to be confirmed^16^. In contrast, an independent UC study showed expansion of a IL26-expressing subset of Th17-like CD8+ T cells with an immunoregulatory signature^89^. Trajectory and TCR sequencing analysis of the single cell profiles further characterised this population as clonally expanded and arising from tissue-resident T cells or representing a post-effector state. To understand the significance of IL26 expression by these cells during inflammation the authors compared pathology of dextran sodium sulfate (DSS)-induced acute colitis in wild-type mice to humanised *IL26*-expressing transgenic mice - the *Il26* gene does not naturally exist in rodents^89^. The IL26 transgenic mice experienced less severe disease, a phenotype that could be reversed with the administration of an IL26-antibody^89^. This suggests a possible role for IL26 in protecting against inflammation. In the context of colorectal carcinoma, paired TCR and transcriptome sequencing identified 8 distinct populations of CD8+ T cells, with signatures ranging from naive, central and effector memory cells, recently activated effector memory/effector cells (TEMRA; with *PRF1*, *GZMB* and *GZMH* expression) to dysfunctional exhausted cells (expressing *PDCD1* and *HAVCR2*)^100^. Distinct TCR clonal populations and trajectory mapping supported two possible differentiation paths for T effector memory cells- either towards TEMRA or exhausted T cell states. The authors suggest that skewing differentiation towards beneficial TEMRA and away from a state of exhaustion could represent a possible avenue for therapeutic intervention^100^. Furthermore, this study showed tumour-specific T cell responses, with enrichment and clonal expansion of pro-inflammatory *CXCL13*+ *BHLHE40*+ TH1-like cells in tumours with microsatellite instability, but moderate enrichment for Th17 cells in those with microsatellite stability^100^. The enrichment of *CXCL13*+ T cells in tumours with high mutational burden was supported by a second scRNAseq study and offers a possible explanation for why this patient cohort responds better to checkpoint blockade therapy^100,101^. Together these studies highlight how scRNAseq can assist in understanding the complexity of intestinal diseases and the nuanced involvement of cell types and states in disease progression and control.

## Zonation of intestinal immunity

Immune cells do not act in isolation, rather their phenotype and response is shaped by their local environment. The intestinal tract in particular comprises unique microenvironments at the macro-anatomical level in terms of distinct tissue regions and at the microanatomical level within the cross-sectional layers of the intestinal wall. Single-cell transcriptomics studies have built upon knowledge of zonation of cells within the human and mouse intestinal tract through providing the full breadth of molecular profiles of cell states, suggesting distinct roles for cells between different zones and how these contribute to the physiological functions of the intestines.

### Zonation between anatomical gut regions

**Fig. 3:**
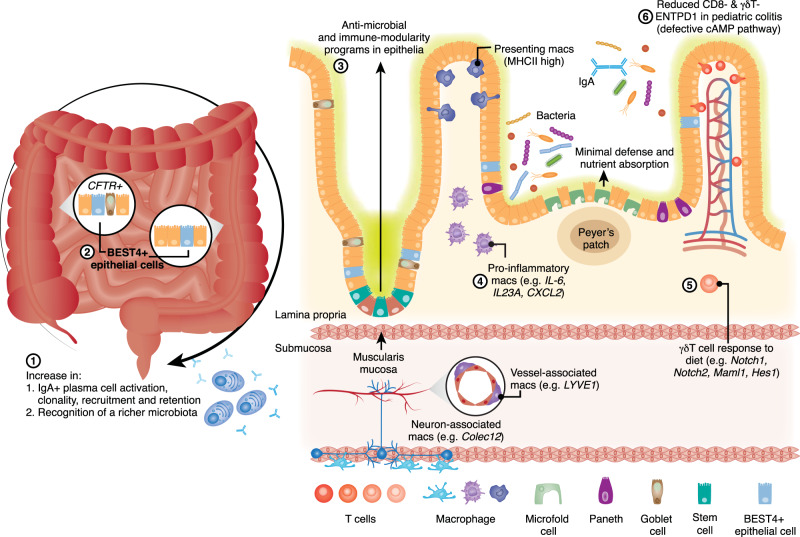
Key scRNAseq finding of zonation of intestinal immunity. (1) ScRNAseq of distinct anatomical regions of the human intestines has revealed increasing activation of plasma cells linked to recognition of a richer microbiota from proximal to distal colon^20^ and (2) small intestine-specific expression of *CFTR* by BEST4+ epithelial cells^21,109^. (3) At the microanatomical level, scRNAseq has shown distinctions in gene expression of the epithelium, with cells at the villus tip expressing immune-modulatory genes^114^ and follicle-associated cells prioritising efficient sampling of microbial antigens^132^. (4) Unbiased clustering of single intestinal macrophages has revealed their unique signatures within the layers of the intestinal wall indicative of specific functions and the influence of interactions with tissue-resident cells^113^. (5) ScRNAseq of physically separated intraepithelial and lamina propria γδ T has implicated the latter in supporting intestinal epithelium remodelling in mice due to change in diet^115^. (6) In the context of colitis, scRNAseq has revealed decreased abundance of CD8- and γδ-ENTPD1 cells in the intraepithelial space and implicated defective cAMP signalling in disease pathogenesis^116^.

With roles in segregation of luminal contents and gut tissue and mediating absorption of nutrients and transfer of signals^102^, the epithelial barrier cells have notable variability between small and large intestines. For example, small intestinal epithelium forms villi and crypts while large intestines form only crypts, and Paneth cells that secrete antimicrobial peptides are only present in the small intestine^103^, while mucus secreting goblet cells are more abundant in the large intestine where they maintain a thicker mucus layer^104^. Through unbiased analysis of gene expression, scRNAseq has shown variability in the expression of nutrient absorption and antimicrobial defence genes by the epithelia between small and large intestines, leading to identification of a Paneth-like cell in the latter^19^. ScRNAseq has also resolved further rare subtypes based on distinct gene expression and shown that these change by gut regions. BEST2+ goblet cells that are restricted to the colon^105,106^, have been deeply profiled at single cell level in humans. This analysis revealed their specific expression of Kallikreins *KLK15* and *KLK3*, and protease inhibitors *WFDC2* and *WFDC3* compared to other colonocytes^21^. Similarly BEST4+ epithelial cells, first identified in the intestinal tract by Ito et al.^105^, have been shown to be transcriptionally distinct from other epithelial cells in the human intestines^15,16^. Building on previous work in which a rare subset of small intestinal epithelial cells was reported to highly express *CFTR*, encoding a key channel mutated in cystic fibrosis^107,108^, further scRNAseq studies showed that BEST4+ epithelial cells of the human small, but not large, intestine co-expressed *CFTR* (Fig. 3)^21,109^. Based on their transcriptional profile and co-localisation with goblet cells, it has been proposed that these cells specifically in the upper intestinal tract support mucus secretion^21,109^ and could be implicated in intestinal symptoms experienced by many cystic fibrosis patients^110^.

Zonation in plasma B cells is similarly described between small and large intestines in humans, with previous studies describing a dominance of IgA1 isotype in small intestine versus IgA2 in the large intestine and an overall trend toward greater abundance of dimeric IgA plasma cells in the latter^111^. A recent single-cell study looked more closely at how these cells changed within the healthy human colon^20^. ScRNAseq of multiple colonic regions from the same individuals not only showed the increasing abundance of IgA+ plasma cells from proximal to distal colon, but transcriptional signatures suggesting this was at least in part due to increased retention/recruitment (Fig. 3). BCR repertoire analysis of the same cells indicated that distal colonic plasma cells were also more clonally expanded and somatically mutated, demonstrating the wealth of information that can be simultaneously obtained through scRNAseq approaches. Paired analysis of the neighbouring microbiota linked the increasing gradient of plasma cell response to recognition of a richer microbiota^20^.

### Zonation at the microanatomical level

The intestinal mucosa can be divided into three compartments- epithelium, lamina propria and muscularis mucosae^112^. These layers are colonised by distinct communities of cells, with substantial interaction and movement between them. The majority of intestinal CD8+ and γδ T cells exist within the intraepithelial layer, while CD4+ T cells typically reside in the lamina propria. Separating these two compartments prior to scRNAseq processing has revealed further surprising details of the zonation of these cell types^113,114^ and their adaptations^115^ and contributions to disease^116,117^.

A study by Sullivan et al. of the mouse small intestinal epithelium showed up-regulation of enteric and pancreatic genes involved in digestion and absorption in response to a high-carbohydrate diet^115^. This gene program was defective in mice depleted of γδ T cells. Following this observation, the authors performed scRNAseq on sorted intraepithelial and lamina propria γδ T cells and identified four transcriptionally distinct populations across both compartments. Surprisingly, while cells of the intraepithelial space would have better access to the epithelium and luminal content, it was γδ T of the lamina propria with the necessary transcriptional profile (i.e., *Notch1*, *Notch2*, *Maml1* and *Hes1*) to permit communication with the epithelium and support its remodelling in response to diet (Fig. 3)^115^. In human coeliac disease, scRNAseq has shown reorganisation of the lamina propria lymphocytes, with natural killer cells of this compartment during health, completely absent during disease^117^. While results of both studies required further validation, they point to finer grain variability of immune cells between intestinal compartments.

ScRNAseq has similarly been applied to better resolve the compartmentalisation of T cells and expression of known risk factors^118^ during Crohn’s disease (CD). Th17 cells and their cytokines are known to be key mediators of the pathogenesis of CD^119^. ScRNAseq has further shown that Th17 cells accumulate within the intraepithelial space at the expense of CD8+ T, γδ T, Tfh and T regulatory cells during active CD compared to controls^120^. Studies of pediatric colitis reported a decreased abundance of CD8-*ENTPD1* (expressing the gene encoding CD39) and γδT-*ENTPD1* cells in the intraepithelial compartment^116^. The transcriptional profile of these specific cell subsets led the authors to hypothesise that a defective cAMP pathway was at play and contributing to disease pathogenesis. To test this theory, the phosphodiesterase inhibitor and anti-platelet drug, dipyridamole, was used to drive the cAMP pathway in a mouse model of colitis and in patients, resulting in a dose-dependent increase in T cell *CD39* expression and improved epithelial integrity and decreased colitis severity^116^.

Separate populations of macrophages exist within the lamina propria, submucosa and muscularis propria. A wealth of earlier research has described diverse roles for these populations appropriate to their microenvironment- lamina propria macrophages phagocytose bacterial antigens and produce mediators that drive epithelial cell renewal and muscularis macrophages interact closely with the enteric nervous system^121^. Bulk RNA sequencing (RNAseq) analysis of macrophages from these physically separated compartments showed separate expression profiles^122^. However, while fluorescence microscopy of mouse intestinal tissue highlighted at least two morphologically distinct populations of muscularis macrophages^122^, the nature and origins of further subsets of macrophages within each compartment remained a mystery. An unpublished study by Domanska et al. implemented scRNASeq of adjacent normal colorectal cancer tissues to address these questions^113^. They showed that lamina propria macrophages comprise 13 transcriptionally distinct subsets with a spectrum of proinflammatory signature (*IL-1B, IL-1A, IL-6, IL23A, CXCL2, CXCL3* and *CXCL8* or *CXCL9, CXCL10, CXCL11, IDO1, GBP1, GBP2, GBP4* and *GBP5*) or high antigen presenting and phagocytic capacity (high levels of HLA class II genes and gene ontology pathways enrichment for endocytosis). Trajectory analysis predicted the majority of these subtypes arise from bone marrow-derived monocytes^113^. In the submucosal space, the majority of macrophages expressed *LYVE1* (associated with vasculature) and *COLEC12* (associated with neurons) and had low antigen presenting capacity, but high chemotactic and tissue-protective properties (Fig. 3). Twelve transcriptionally distinct populations of macrophages were present in the muscularis propria with proinflammatory properties (e.g., expression of *IL1A*, *IL1B*, *CXCL2*, *CXCL3*, *CXCL8*, *CCL3* and *CCL4*) and homeostatic properties (e.g., expression of *LILRB5*, *MARCO*, *LYVE1*, *FOLR2* and *COLEC12*). Homeostatic muscularis macrophages were also positive for *PMP22* and *EMP1*, genes expressed by Schwann cells, suggesting these macrophages phagocytose Schwann cells and are in close contact with neurons^113^. Macrophages in both compartments showed ligand/receptor expression enabling them to interact extensively with tissue resident cells indicating that their expression profile is heavily influenced by their local microenvironment^113^.

Intestinal epithelial cells arise from a common stem cell at the crypt base and transdifferentiate as they move towards the villus tip. Although the positions of enterocytes along the villus axis correlate with their age^123^, exposure to morphogen gradients^124^, and hypoxia^125^, low-resolution approaches were unable to determine the positional effects on enterocyte function in mice^126,127^ or humans^128^. A study by Moor et al. applied laser capture microdissection of mouse enterocytes followed by scRNAseq to elegantly resolve a continuous gradient of transdifferentiation along the villus axis^114^. The villus tip enterocytes expressed an immune-modulatory program with the capacity to modulate immune reaction to the microbiota in the gut lumen (Fig. 3)^114^. Follicle-associated epithelium covering the lymphoid structures (i.e., PPs) possess characteristics distinct from villus epithelium^129–131^. Microdissection and RNAseq of mouse intestinal epithelium followed by single-cell validation of gene expression with single molecule fluorescence in situ hybridization showed that follicle-associated epithelium expresses lower levels of antimicrobial and nutrient absorption genes^132^. This suggests that epithelium at these sites is tuned for the optimal and efficient sampling of bacterial antigens by M cells and immune cells, rather than nutrient absorption and antimicrobial activity (Fig. 3).

## Intestinal immunity shaped by non-immune interactions

Key components of tissue microenvironments are the resident non-hematopoietic cells that have multiple established roles in immune responses and inflammation in mucosal surfaces. While previously this involvement was thought to be passive, with research focusing on fibrosis, tumour progression and wound healing, scRNAseq studies are highlighting the extent of active engagement of non-immune cells in shaping mucosal immunity with implication for health and disease progression^133,134^.

Mesenchymal or stromal cells of the intestine reside in the subepithelial layers and contribute largely to structural integrity. Three recent studies comparing healthy and IBD intestinal tissue have applied scRNAseq to not only map the diversity of intestinal stromal subtypes, but also pinpoint which cell subtypes and interactions are at play during inflammation^16–18^. Kinchen et al. defined four distinct stromal populations with unique transcriptional signatures^17^. One of these stromal types termed stromal 4 cells, marked by expression of genes involved in cytokine signalling, T cell activation and cell adhesion, was scarce in healthy controls and enriched in UC. Crucially, *IL-6* and *TNFSF14* were additionally expressed by stromal 4 cells during disease and shown to prevent epithelial regeneration in follow up intestinal organoids experiments. Martin et al. similarly observed stromal cells contributing to the cellular response of CD^18^. However, here they defined a collective cell module (termed GIMATS) consisting of IgG plasma cells, inflammatory mononuclear phagocytes, activated T cells, and stromal cells that corresponded with failure to achieve durable corticosteroid-free remission upon anti-TNF therapy in a fraction of patients. A real strength of scRNAseq here was the capacity to compare ligand receptor pair expression between equivalent cells of patient groups. In this way, the authors showed that enriched cellular interactions between myeloid cells, activated endothelial cells and activated *CCL2*+ *CCL7*+ stromal cells were generating a positive inflammatory feedback loop in the GIMATS samples^18^. Last, Smillie et al. identified a population of inflammation associated fibroblasts (IAFs) that were expanded 189-fold in biopsies from UC patients versus controls^16^. The profile of IAFs was comparable to cancer-associated fibroblasts, a key player in creating an immune tolerant tumour environment. IAFs also highly express *OSMR*, a predictor of resistance to anti-TNF therapy in UC patients, and ranked high for a resistance gene signature determined from bulk RNAseq data. The gene encoding the ligand for OSMR, *OSM* was most strongly expressed by inflammatory monocytes and cDC2s in the scRNAseq data, implicating interactions between these cells in resistance to treatment^16^.

Tuft cells are chemosensors of the gut epithelium, transmitting messages in the form of a spectrum of biological effector molecules to immune and neuronal cells^135^. Previous bulk RNAseq had identified neuronal and inflammation gene signatures from these cells^136^, but was unable to resolve whether these programs were from one population of cells or distinct subtypes. Using scRNAseq Haber et al. carried out unbiased clustering of Tuft cells from the small intestines of mice and identified two distinct subsets contributing these profiles^137^. Tuft-2 cells were enriched for immune-related genes particularly those supporting Th2 responses (*Il4ra* and *Il13ra1* and *Il17rb*). Incredibly, this population also expressed Ptprc (encoding the pan-immune marker CD45), the first recording of this in non-hematopoietic cells and blurring the lines of the traditional definition of immune cells^137^. While equivalent findings have not been made from single cell analysis of human intestinal tuft cells, a fraction of human and mouse Tuft cells were shown to express immune signalling machinery, specifically activating and inhibitory Fc gamma receptors and downstream mediators for IgG signalling^21^. This could facilitate direct activation of Tuft cells in response to signals from plasma cells. These findings formed the basis of experiments in mouse models of intestinal colitis in which Tuft cells upregulated the inhibitory receptor suggested their potential as a rheostat of intestinal inflammation^21^.

Antigen presentation is a critical step in the transmission of immune activation to the adaptive immune system with primary antigen presenters regarded as conventional DCs, macrophages and naive B cells. A body of prior work has extended this role to various epithelial cell types via MHC-II expression, with particular roles for microfold cells localised to PPs^138^. Recent scRNAseq experiments have taken this further to pinpoint exact subpopulations. Work from the Xavier and Regev laboratories combined scRNAseq, flow cytometry and immunofluorescence assays to define three novel subtypes of Lgr5+ intestinal stem cells in the mouse small intestine^82^. Although not as high as DCs, two of these populations expressed MHC-II at significant levels and were capable of presenting antigen to antigen-specific T cells in co-culture experiments. While the exact role of antigen presentation by these cells is unknown, the authors speculated that it could be a non-essential means for the epithelial layer to respond to infection or be a means by which T helper cells can interact with ISCs and shape their appropriate differentiation into mature epithelial cell types. The latter explanation is particularly interesting in light of further results showing Tregs cells promote ISC renewal while Th1 and Th17 cells promote differentiation^82^.

## Concluding remarks and future perspectives

Single-cell studies have provided a wealth of knowledge about the complex cellular landscape of the intestinal immune system. In their short history, they have detailed the spectrum of cell phenotypes, provided resolution of zonation of immune cells and shown the impact of their engagement with the neighbouring microbiota in health and disease. As these methods continue to evolve, there is little doubt that they will continue to provide insights into the field of intestinal immunity (Box 1).

Spatial transcriptomics, while not yet at the single-cell with whole transcriptome level, has already placed gut immune signatures in their tissue context^21,24^ and will be a key feature of future studies. The application of scRNAseq to in vitro systems and experimental models will offer the ability to look in detail at the mechanism of therapeutic and biological agents (e.g., faecal microbiota) on intestinal immune cells. ScRNAseq has already been adapted for capture of bacterial RNA in pioneering studies^139^, opening the possibility to study the function of specific bacteria. Integration of modalities for example combining scRNAseq of host cells with single-cell metatranscriptomics and patient genotype data will also provide the opportunity to study the interaction between these factors in shaping intestinal immune environments. Last, as the first chapter of the HCA approaches completion^140^, studies of individual organ systems will be combined to provide a global picture of human biology. We anticipate that this will bring with it studies of the contribution of gut immune cells to human biology and disease at a systems-wide level.

Box 1 Areas of open investigation
