# Correction to: Redefining intestinal immunity with single-cell transcriptomics

**DOI:** 10.1038/s41385-022-00483-1

**Published:** 2022-02-07

**Authors:** Kylie Renee James, Rasa Elmentaite, Sarah Amalia Teichmann, Georgina Louise Hold

**Affiliations:** 1 Garvan-Weizmann Centre for Cellular Genomics, Garvan Institute of Medical Research, Sydney, NSW Australia; 2 School of Medical Sciences, University of New South Wales, Sydney, NSW Australia; 3 Wellcome Sanger Institute, Hinxton, UK; 4 Theory of Condensed Matter Group, Cavendish Laboratory/Department of Physics, University of Cambridge, Cambridge, NSW UK; 5 University of New South Wales Microbiome Research Centre, Sydney, NSW Australia

Correction to: *Mucosal Immunol* 10.1038/s41385-021-00470-y, published online 30 Nov 2021.

The original version of this article unfortunately contained a mistake. Due to a typesetting error Box 1 was omitted. We apologize for the error. Box 1 can be found below. The original article has been corrected.

